# Enhancement of Mitochondrial Function by the Neurogenic Molecule NSI-189 Accompanies Reversal of Peripheral Neuropathy and Memory Impairment in a Rat Model of Type 2 Diabetes

**DOI:** 10.1155/2022/8566970

**Published:** 2022-07-04

**Authors:** C. G. Jolivalt, M. R. Aghanoori, M. C. Navarro-Diaz, M. M. Han, G. Sanchez, L. Guernsey, D. Quach, K. Johe, P. Fernyhough, N. A. Calcutt

**Affiliations:** ^1^University of California San Diego, Department of Pathology, La Jolla, CA, USA; ^2^Division of Neurodegenerative Disorders, St. Boniface Hospital Albrechtsen Research Centre, Winnipeg, MB, Canada; ^3^Department of Pharmacology and Therapeutics, University of Manitoba, Winnipeg, MB, Canada; ^4^Neuralstem Inc., Germantown, MD, USA

## Abstract

**Aims:**

Mitochondrial dysfunction contributes to many forms of peripheral and central nervous system degeneration. Therapies that protect mitochondrial number and function have the potential to impact the progression of conditions such as diabetic neuropathy. We therefore assessed indices of mitochondrial function in dorsal root ganglia (DRG) and brain cortex of the Zucker diabetic fatty (ZDF) rat model of type 2 diabetes and tested the therapeutic impact of a neurogenic compound, NSI-189, on both mitochondrial function and indices of peripheral and central neurological dysfunction.

**Materials and Methods:**

ZDF rats were maintained for 16 weeks of untreated diabetes before the start of oral treatment with NSI-189 for an additional 16 weeks. Nerve conduction velocity, sensitivity to tactile and thermal stimuli, and behavioral assays of cognitive function were assessed monthly. AMP-activated protein kinase (AMPK) phosphorylation, mitochondrial protein levels, and respiratory complex activities were assessed in the DRG and brain cortex after 16 weeks of treatment with NSI-189.

**Results:**

Treatment with NSI-189 selectively elevated the expression of protein subunits of complexes III and V and activities of respiratory complexes I and IV in the brain cortex, and this was accompanied by amelioration of impaired memory function and plasticity. In the sensory ganglia of ZDF rats, loss of AMPK activity was ameliorated by NSI-189, and this was accompanied by reversal of multiple indices of peripheral neuropathy.

**Conclusions:**

Efficacy of NSI-189 against dysfunction of the CNS and PNS function in type 2 diabetic rats was accompanied by improvement of mitochondrial function. NSI-189 exhibited actions at different levels of mitochondrial regulation in central and peripheral tissues.

## 1. Introduction

Mitochondrial dysfunction and subsequent cellular energy imbalance contribute to many forms of neurodegenerative disease. Hereditary mutations to mitochondrial DNA cause the syndrome known as mitochondrial encephalopathy, lactic acidosis and stroke-like episodes (MELAS) [1] while slowly developing disorders such as Alzheimer's disease [2], Parkinson's disease [3], Huntington's disease [4], and amyotrophic lateral sclerosis [5] are also associated with aberrant mitochondrial regulation and function. Mitochondrial dysfunction also contributes to the pathogenesis of peripheral neuropathies triggered by metabolic, chemotoxic, or infectious insults [6, 7]. Peripheral neuropathy has long been recognized as a debilitating complication of diabetes [8]. Physical damage to the central nervous system (CNS) has also been documented in autopsy reports for over half a century [9], but only more recently has cognitive dysfunction with increased risk of developing dementia and Alzheimer's disease been widely recognized in patients with type 1 [10] or 2 [11, 12] diabetes. Therapies that protect mitochondrial number and function or enhance activity of residual mitochondria have the potential to impact diverse neurological conditions for which there are few treatment options.

NSI-189 is an orally active molecule that stimulates synaptogenesis, neurogenesis, and increased hippocampal volume in models of stroke, Angelman syndrome, and irradiation-induced brain injury [13]. Clinical trials in subject with major depressive disorder (MDD) demonstrated that NSI-189 is safe and had long-lasting antidepressive and procognitive effects [14, 15]. We recently reported that NSI-189 prevented and reversed memory impairments, restored hippocampal neurogenesis, and enhanced expression of synaptic markers in the CNS of type 1 or type 2 diabetic mice while concurrently ameliorating peripheral neuropathy [16]. Intriguingly, NSI-189 applied to sensory neurons in vitro both enhanced neurite outgrowth and stimulated maximal and spare respiratory capacity [16]. The present study expands investigation of the ability of NSI-189 to stimulate mitochondria and ameliorate indices of CNS and PNS dysfunction in the Zucker diabetic fatty (ZDF) rat model of type 2 diabetes.

## 2. Materials and Methods

### 2.1. Animals

All protocols were approved by the Institutional Animal Care and Use Committee of the University of California, San Diego. Experiments were performed in adult male ZDF rats, a model of obese type 2 diabetes, and age- and sex-matched lean control rats (strains 370 and 371, Charles River Laboratories, CA, USA). Rats were housed 2/cage with free access to water and food (5001 PMI diet, Harlan, Indianapolis, USA). ZDF rats carry a spontaneous mutation of the leptin receptor and develop obesity and insulin resistance at 8-10 weeks of age followed by progressive development of hyperglycemia. Daily treatment with vehicle or NSI-189 (30 mg/kg po) was initiated at 24 weeks of age (16 weeks of diabetes) and continued for 16 weeks.

### 2.2. Glucose Tolerance Test

The glucose tolerance test was performed, before the start of treatment and after 12 weeks of treatment, after an overnight fast by intraperitoneal injection of 1.5 g glucose/kg body weight. Glucose levels were measured every 30 min for 2 hours in a blood sample obtained by tail prick using a test strip and meter (One Touch Ultra, Lifescan Inc. Milpitas, CA, USA).

### 2.3. Drugs

NSI-189 phosphate ((4-benzylpiperazin-1-yl)-[2-(3-methylbutylamino)pyridin-3-yl] methanone) was provided by Neuralstem (now Palisade Bio Inc., CA, USA) and dissolved in 0.2 N HCl in distilled water (10/90 *v*/*v*, pH 4-5). NSI-189 or vehicle (0.2 N HCl/water, pH 4-5) was administered daily by oral gavage. Unless noted, assays were performed 24 h after the most recent treatment to preclude detection of transient effects.

### 2.4. Object Recognition Test

The rat was first placed in a box and presented with two similar objects. The selection criteria required each rat spending 15 s exploring both objects. One hour later, one of the objects was replaced by a new object. The amount of time taken to explore the new object (t2), relative to the time spent on the first object (t1), provided the %recognition index = ((t2–t1)/(t1 + t2)) × 100 [17].

### 2.5. Tactile Responses

Paw responsiveness to light touch was measured with von Frey filaments as described previously [18]. The mean 50% paw withdrawal threshold (50% PWT) of both hind paws represented each animal [19].

### 2.6. Thermal Responses

Paw withdrawal to heating (1°C/s) was measured by placing rats in the observation chamber of a thermal testing apparatus (UARD, CA, USA). Response latency of both paws was tested 3 times at 5 min intervals, the median response time for each paw calculated, and the mean of the two medians used to represent each rat [19].

### 2.7. Electrophysiology

Motor and sensory nerve conduction velocity (MNCV and SNCV, respectively) were measured in the sciatic nerve : interosseous muscle system of isoflurane-anesthetized rats [20]. Using an AD Instruments Powerlab 4/30 (AD Instruments, CO, USA), the sciatic nerve was stimulated (1-5 V, 0.05 ms square wave) via needle electrodes placed at the sciatic notch or ankle to deliver a single supramaximal stimulus. *M* and *H* waves were measured in the resulting electromyograms with the median of each triplicate used to calculate MNCV and SNCV following measurement of the distance between the two stimulation sites.

### 2.8. Corneal Nerve Visualization and Quantification

Rats were anesthetized with isoflurane and placed on the imaging platform of a Retina Tomograph 3 with Rostok corneal module (Heidelberg Engineering, Heidelberg, Germany). Corneal images were collected at 2 *μ*m intervals [21], and total nerve length present in 5 sequential images of the subbasal plexus and 10 sequential images of the stroma was calculated using the ImageJ software.

### 2.9. Epidermal Nerve Visualization and Quantification

Hind paw plantar skin was removed at autopsy into 4% paraformaldehyde for 24 hours. Skin samples were embedded in paraffin and cut as 6 *μ*m sections before incubation with an antibody against the pan-neuronal marker PGP9.5 (1 : 1000, Biogenesis Ltd., UK). The number of epidermal PGP9.5 immunoreactive profiles per unit length of tissue was quantified by light microscopy [22].

### 2.10. Hippocampal Volume

One brain hemisphere was removed into 4% paraformaldehyde and the other dissected into the hippocampus and cortex. Tissues were frozen and stored for Western blot analysis. Hippocampal sections (40 *μ*m) were viewed using a Nikon AZ100 light microscope and hippocampal volume measured by tracing [23].

### 2.11. Protein Expression in the Rat Hippocampus, Cortex, and DRG

Hippocampi were homogenized in buffer containing 50 mM Tris-HCl pH 7.4, 150 mM NaCl, 0.5% Triton X, 1 mM EDTA, and protease inhibitor cocktail. Five to 20 *μ*g of total proteins were processed and separated on 4-12% SDS-PAGE Bis-Tris gels (Novex, Invitrogen, Carlsbad, CA, USA) before being transferred to nitrocellulose as described in detail elsewhere [24]. Blots were incubated with antibodies against NeuN (1 : 1000, Millipore, USA), synaptophysin (1 : 10000, Millipore, USA), PSD95 (1 : 500, Chemicon, USA), or actin (1 : 5000, Sigma, USA) followed by incubation with corresponding secondary antibodies tagged with infrared dyes (IRDye, 1 : 15000, LI-COR Biosciences, Lincoln, NE, USA). For each animal, band intensities were normalized by calculating the ratio of the intensity of bands corresponding to the antigen of interest to the intensity of the band corresponding to actin. The actin-normalized data for each lane were expressed as a percentage of the group mean of values obtained from control rats.

The DRG and whole brain cortex were homogenized in cold RIPA buffer (25 mM Tris pH = 8, 150 mM NaCl, 0.1% SDS, 0.5% sodium deoxycholate, 1% Triton X-100, and protease phosphatase inhibitors). Seven and half to 10 *μ*g of total protein were separated on a 10% SDS-PAGE gel and transferred to the nitrocellulose membrane. Blots were incubated with antibodies to phosphorylated (Thr172) AMPK (pAMPK; 1 : 500, Santa Cruz Biotechnology Inc., Santa Cruz, CA) and total AMPK (T-AMPK; 1 : 500, Cell Signaling Technology, Boston, MA). Respiratory chain subunits were detected using an antibody cocktail (Invitrogen, 1 : 1000 dilution) that detected complex I, NDUFB8; complex II, SDHB; complex III, UQCRC2; complex IV, MTCO1, and complex V, ATP5a. Total extracellular-regulated protein kinase (T-ERK; 1 : 2000, Santa Cruz Biotechnology) was used as a loading control as T-ERK expression does not change in either DRG or neuronal cultures obtained from diabetic rodents. Densitometry of the resulting blots was performed using an image analyzer (ChemiDoc, Bio-Rad, CA, USA).

### 2.12. Respiratory Complex Activities in the Cortex and DRG

Enzymatic activity of complex IV and of cytochrome *c* oxidase was measured in rat cortex homogenates using a temperature-controlled UV-visible spectrophotometer (Ultrospec 2100, Biopharmacia Biotech, Uppsala, Sweden) and Biochrom Swift II software as previously described [25]. Mitochondrial complex I enzymatic activity was measured using a colometric assay (Cat #:K968-100, BioVision, CA, USA) and UV-visible spectrophotometer. Due to the limited tissue available, only enzymatic activity of complex IV was measured in DRG tissue samples.

### 2.13. Plasma Analysis

Plasma obtained at termination of the study was assessed for insulin levels using the rat insulin ELISA, Mercodia AB, Uppsala, Sweden.

### 2.14. Data Analysis

All animals and tissues were coded to preclude observer bias. Data are presented as group mean ± SEM. Statistical comparisons were made by one-way ANOVA with between-group differences identified using Tukey's or Holm-Sidak *post hoc* tests or repeated measures ANOVA with between-group differences identified using Holm-Sidak or Tukey's *post hoc* test.

## 3. Results

### 3.1. Diabetes

ZDF rats at 24 weeks of age (onset of treatment) showed significant obesity, hyperglycemia, hyperinsulinemia (Supplemental Table 1S), and impaired glucose tolerance (Supplemental Figure 1SA). Sixteen weeks of daily treatment with NSI-189 did not affect blood glucose or reduced insulin levels at 40 weeks of diabetes (Supplemental Table 1S, Supplemental Figure 1SB).

### 3.2. PNS

#### 3.2.1. Nerve Conduction Velocity

**Figure 1:**
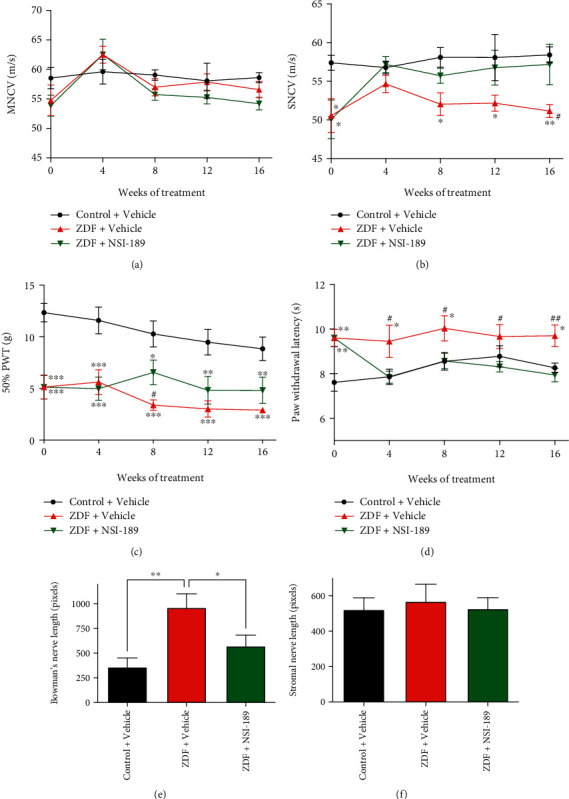
Functional indices of diabetic neuropathy were reversed by NSI-189: (a) motor nerve conduction velocity and (b) sensory motor nerve conduction velocity, (c) tactile responses to von Frey filaments, and (d) thermal responses on the Hargreaves apparatus, over the 16 weeks of treatment with NSI-189. ^∗^*p* < 0.05, ^∗∗^*p* < 0.01, and ^∗∗∗^*p* < 0.001, two-way repeated measures ANOVA followed by Tukey's *post hoc* test against the control+vehicle group; ^#^*p* < 0.05, ^##^*p* < 0.01 against the ZDF+NSI-189 group. Black circle: control+vehicle, red upward triangle: ZDF+vehicle, and green downward triangle: ZDF+NSI-189 groups. (e) Corneal nerve length in Bowman's subbasal layer and (f) in the stromal layer. ^∗^*p* < 0.05, ^∗∗^*p* < 0.01, one-way ANOVA followed by Holm-Sidak's *post hoc* test against the ZDF+vehicle group. *N* = 10 animals per group, except for CCM data of control rats where the quality of 2 sets of images was not satisfactory for analysis. Data are mean ± SEM.

MNCV was not different between groups at any time (Figure 1(a)). Prior to onset of NSI-189 treatment, ZDF rats exhibited significant (*p* < 0.05 vs. vehicle-treated control rats) SNCV slowing (Figure 1(b)). NSI-189 restored SNCV in ZDF rats to the level of control rats, and this was maintained for the duration of the study (Figure 1(b), *p* < 0.05 vs. vehicle-treated ZDF rats).

#### 3.2.2. Tactile Sensation

Prior to treatment with NSI-189, ZDF rats showed significant (*p* < 0.001) tactile allodynia (Figure 1(c)). Vehicle-treated ZDF rats showed progressive worsening of allodynia over time that was halted by NSI-189 treatment so that there was a significant (*p* < 0.05) difference from vehicle-treated ZDF rats at week 8, although both groups remained significantly allodynic compared to controls.

#### 3.2.3. Heat Sensation

Before onset of treatment with NSI-189, ZDF rats showed significant (*p* < 0.01) thermal hypoalgesia (Figure 1(d)). After 4 weeks of treatment with NSI-189, hypoalgesia was significantly (*p* < 0.05) reversed and thermal response latencies of NSI-189-treated ZDF rats remained similar to those of control rats for the rest of the study (Figure 1(d)).

#### 3.2.4. Small Fiber Density

At the study end, intraepidermal nerve fiber (IENF) density in plantar footskin showed a mild trend to reduction in ZDF rats (control: 28.3 ± 2.6, ZDF: 25.4 ± 1.3 IENF/mm) that was not impacted by NSI-189 treatment (ZDF+NSI-189: 24.25 ± 1.8 IENF/mm). In contrast, small sensory fiber density in the corneal subbasal nerve plexus of vehicle-treated ZDF rats showed a significant (*p* < 0.01) increase in nerve density compared to vehicle-treated lean rats that was partially normalized by 16 weeks of NSI-189 treatment (Figure 1(e)). No differences between groups were observed in small sensory nerves of the corneal stroma (Figure 1(f)).

#### 3.2.5. Mitochondrial Protein Expression and Activity

**Figure 2:**
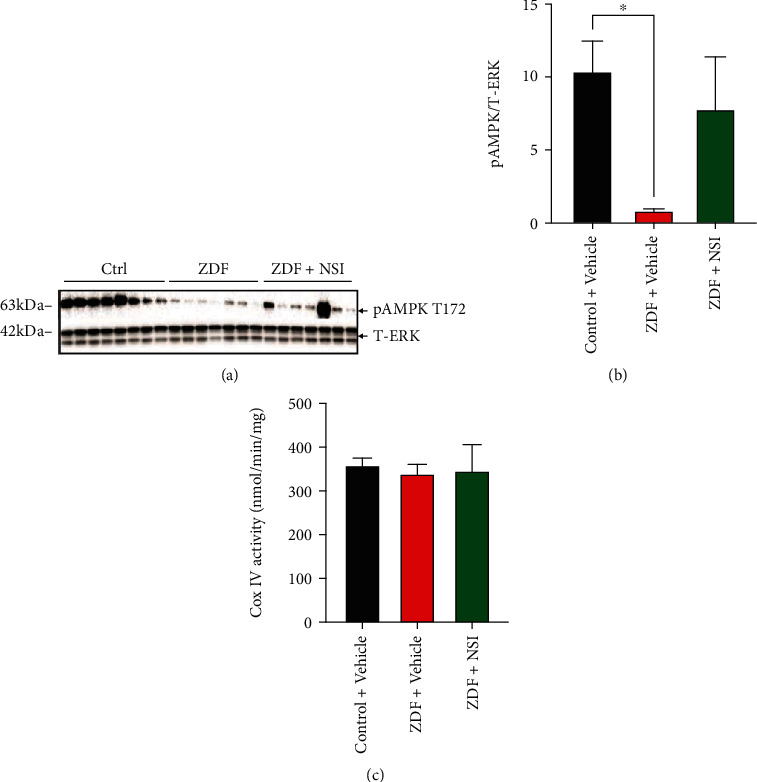
AMPK activity in DRG tissues from ZDF animals: lumbar DRG tissues were obtained from control (Ctrl), ZDF, and NSI-treated ZDF (ZDF+NSI) rats and underwent (a) Western blotting or (c) cytochrome c oxidase activity assay. (a) Phosphorylated AMPK (pAMPK) and total ERK (T-ERK) Western blot image. (b) Intensity of bands shown in (a). (c) Activity of cytochrome *c* oxidase (Cox IV). Data are mean ± SEM of *N* = 7-8 for Western blotting and *N* = 4 for cytochrome *c* oxidase activity assay; ^∗^*p* < 0.05, analyzed by one-way ANOVA with Holm-Sidak's *post hoc* test. Data are mean ± SEM.

The amount of tissue available for DRG restricted the assays performed to measure pAMPK levels and cytochrome c oxidase activity. Phosphorylated AMPK at threonine 172 (pAMPK T172), representing the active enzyme, was significantly (*p* < 0.05) reduced in the DRG of ZDF rats, and treatment with NSI-189 for 16 weeks partially elevated pAMPK levels (Figures 2(a) and 2(b)). Enzyme activity of cytochrome *c* oxidase (complex IV) was not different amongst the 3 groups (Figure 2(c), *N* = 4 per group due to the limited amount of tissue homogenate).

### 3.3. CNS

#### 3.3.1. Memory

**Figure 3:**
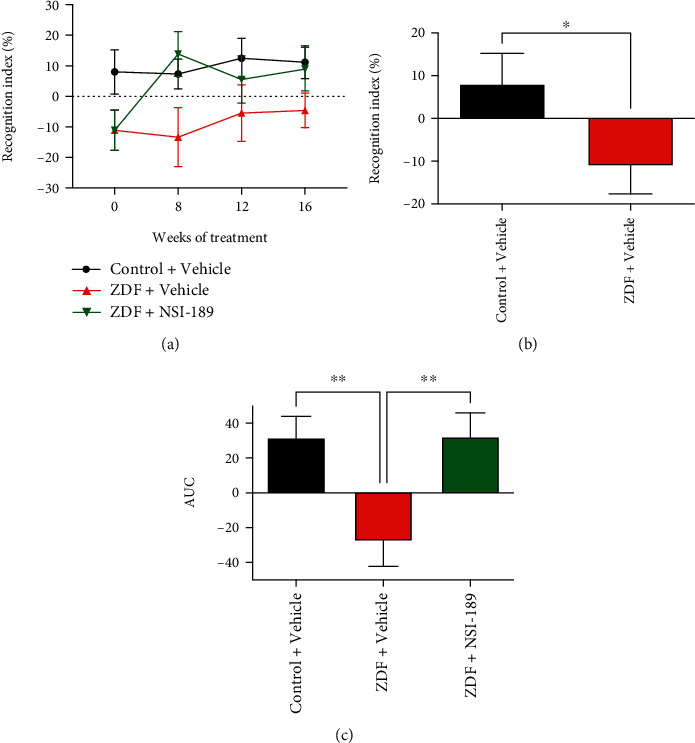
NSI-189 reverses deficits in memory in ZDF rats. (a) Time course of recognition index (%) in the object recognition test over the 16 weeks of treatment with NSI-189. (b) Recognition index (%) in the objection recognition test performed before the start of treatment and (c) area under the curve (AUC) of the curve shown in (a) representing the duration of the study with tests every 4 weeks; ^∗^*p* < 0.05, ^∗∗^*p* < 0.01, analyzed by one-way ANOVA with Holm-Sidak's *post hoc* test. *N* = 10 animals per group. Data are mean ± SEM.

Memory was assessed using the object recognition test (Figure 3) prior to and 8, 12, and 16 weeks after (represented as area under the curve (AUC)) treatment (Figures 3(b) and 3(c)). Short-term episodic memory was significantly (*p* < 0.01) decreased in ZDF rats prior to the start of treatment (Figure 3(b)). Sixteen weeks of treatment with NSI-189 significantly (*p* < 0.01) restored impaired memory of ZDF to memory function similar to that of control rats (Figure 3(c)).

#### 3.3.2. Neuronal Plasticity

**Figure 4:**
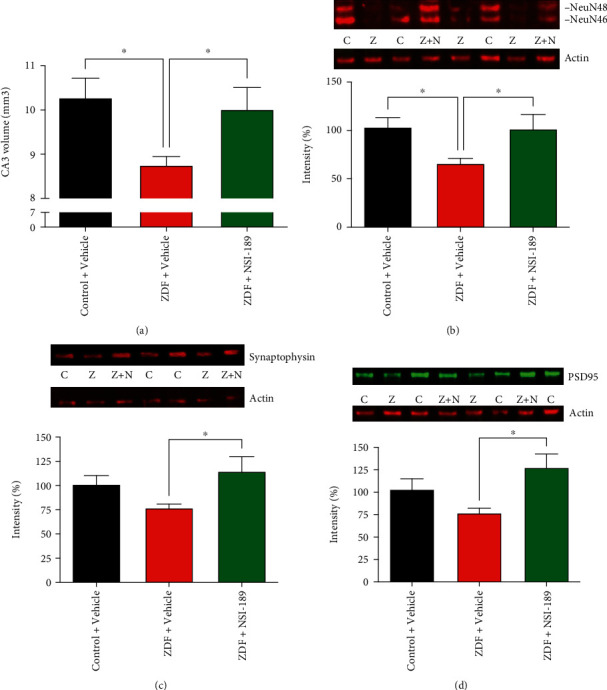
NSI-189 reverses deficits in synaptic plasticity and hippocampal volume. (a) Hippocampal CA3 volume. Western blot analysis for (b) NeuN, (c) synaptophysin, and (d) PSD-95 proteins (C: control, Z: ZDF, and Z+N: ZDF+NSI-189 groups). ^∗^*p* < 0.05, ^∗∗^*p* < 0.01, one-way ANOVA followed by Holm-Sidak's *post hoc* test against the ZDF+vehicle group. *N* = 8-10 animals per group, due to the quality of bands. Data are mean ± SEM.

A significant (*p* < 0.05) decrease in hippocampal volume (CA3 area) of ZDF rats was prevented by NSI-189 (Figure 4(a)), while the neuronal marker NeuN followed the same pattern (Figure 4(b)). Neuronal plasticity was further assessed by expression of the synaptic marker synaptophysin, and the postsynaptic protein PSD95 expression of both proteins trended lower in the hippocampus of ZDF rats while 16 weeks of treatment with NSI-189 significantly (*p* < 0.05) elevated levels of both proteins above those of vehicle-treated ZDF rats (Figures 4(c) and 4(d)).

#### 3.3.3. Cortical Mitochondrial Protein Expression and Activity

**Figure 5:**
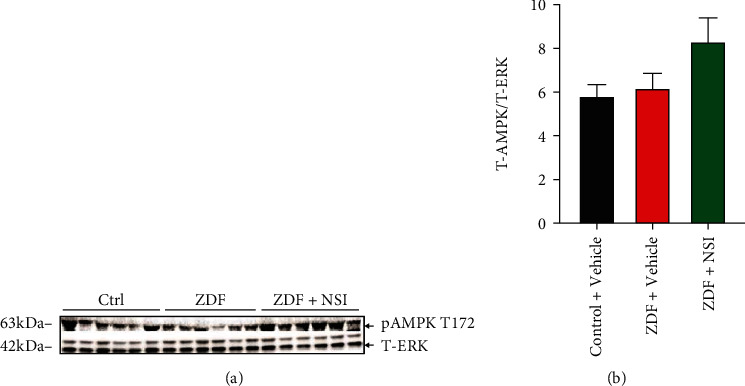
AMPK activity in brain cortex tissues from ZDF animals. The cortex was obtained from control (Ctrl), ZDF, and NSI-treated ZDF (ZDF+NSI) rats and underwent Western blotting for (a) pAMPK and T-ERK signals. (b) Intensity of bands shown in (a). Data are mean ± SEM of *N* = 6.

**Figure 6:**
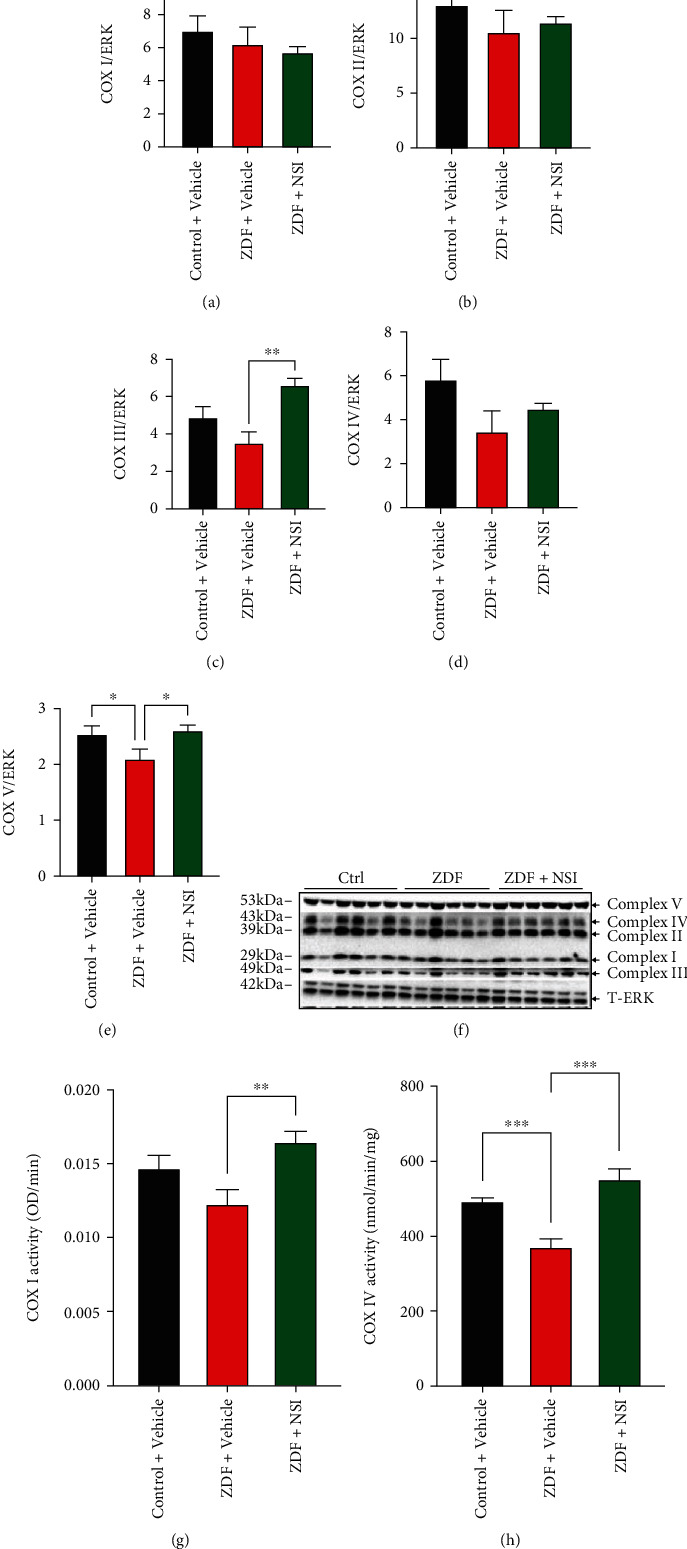
NSI treatment restores mitochondrial complex I and cytochrome *c* oxidase activity in the brain cortex from ZDF rats in parallel with elevation of expression of respiratory chain proteins. Brain cortex tissues were obtained from control (Ctrl), ZDF, and NSI-treated ZDF (ZDF+NSI) rats and underwent (a–f) Western blotting or (g, h) complex I and complex IV activity assays. (b) Protein levels for (a) Cox I (NDUFB8), (b) Cox II (SDHB), (c) Cox III (UQCRC2), (d) Cox IV (MTCO1), and (e) Cox V (ATP5a). (f) Western blot image of subunits of mitochondrial complexes I-V and total ERK (T-ERK). (g) Complex I activity and (h) cytochrome *c* oxidase (Cox IV) activity. Data are mean ± SEM of *N* = 6; ^∗^*p* < 0.05, ^∗∗^*p* < 0.01, and ^∗∗∗∗^*p* < 0.0001, analyzed by one-way ANOVA with Holm-Sidak's *post hoc* test.

To accompany assays of memory and synaptic markers, the brain cortex was processed for analysis of pAMPK and mitochondrial complexes. Phosphorylated AMPK levels were similar in the cortex of control and ZDF rats, and treatment with NSI-189 did not significantly affect AMPK phosphorylation, although there was a trend towards increase (Figures 5(a) and 5(b)). Expression of NDUFB8 (complex I), SDHB (complex II), and MTCO1 (complex IV) subunit proteins was also unchanged in all 3 groups of rats (Figures 6(a), 6(b), 6(d), and 6(f)). In contrast, expression of UQCRC2 (complex III subunit) and ATP5a (complex V subunit) was significantly increased in the cortex of ZDF rats following treatment with NSI-189 (Figures 6(c), 6(e), and 6(f)). Enzyme activity of complexes I and IV decreased in homogenates of the ZDF rat cortex and was significantly (*p* < 0.01) increased by NSI-189 (Figures 6(g) and 6(h)).

## 4. Discussion

Treatment with NSI-189 was initiated after a period of 16 weeks of untreated diabetes to mirror interventional use in patients with long-term type 2 diabetes and established neuropathy. NSI-189 reversed the effects of type 2 diabetes on indices of both small and large fiber peripheral neuropathy along with short-term memory dysfunction. These effects were maintained for the duration of the study and accompanied by enhancement of distinct patterns of mitochondrial function in the PNS and CNS.

ZDF rats develop multiple indices of neuropathy that model the human condition [26, 27]. Following 16 weeks of untreated diabetes, ZDF rats showed large fiber dysfunction as indicated by SNCV slowing and allodynia. Unlike others [26, 27], we did not find large fiber MNCV slowing. We have previously reported a similar exaggeration of SNCV slowing compared to MNCV slowing in ZDF rats [28]. Perikarya of sensory neurons are more exposed to blood-borne toxins, including glucose, than perikarya of motor neurons, due to their location in DRG versus the spinal dorsal horn, and this may contribute to more marked SNCV slowing. SNCV slowing was reversed by daily treatment with NSI-189, while progression of tactile allodynia was halted. Whether recovery of sensory nerve conduction velocity is associated with maintenance of large sensory fiber axonal caliber, which shows atrophy in this model, remains to be determined. There was no evidence of drug tolerance as efficacy of NSI-189 was maintained for the study duration. These findings extend prior data obtained in mouse models of diabetes [16] and emphasize that NSI-189 shows efficacy in multiple species. The ability of NSI-189 to reverse established neuropathy is encouraging, as intervention is the most likely scenario for translation to clinical use, particularly as large fiber conduction slowing and pain are currently accepted readouts for therapeutic efficacy in clinical trials [29].

Measures of small sensory nerve fiber function and structure did not entirely follow patterns anticipated from prior literature. Paw heat hypoalgesia was present in ZDF rats before onset of treatment with NSI-189 and was reversed within 4 weeks, which is consistent with equivalent studies in diabetic mice. However, while previous studies in diabetic mice showed that heat hypoalgesia was accompanied by loss of plantar IENF and that NSI-189 restored both heat sensation and IENF density, the ZDF rats showed only a mild reduction in paw IENF density. A similar mild trend to decrease of paw IENF has been reported in ZDF rats [30], whereas others reported more marked deficits [26, 31]. Survival and/or accelerated regrowth of IENF may reflect increased circulating insulin levels in the early stage, despite decreasing levels in the later stage, as sensory neuron energy balance, neurite outgrowth, and paw IENF density are modulated by insulin, independent of hyperglycemia [32–35]. Moreover, while it is attractive to equate loss of heat sensation to physical depletion of IENF, sensory loss precedes detectable loss of IENF in other models of diabetes [22]. Loss of heat sensation in this study may represent physiological rather than pathological disorders. The most striking divergence from anticipated data was our observation that nerve density in the corneal subbasal plexus was significantly increased in 10-month-old ZDF rats. This increase contrasts with the typical decrease in corneal nerve density seen in models of type 1 diabetes [16, 21, 36, 37], in a model of late type 2 diabetes resulting from feeding a high fat diet combined with a low dose of STZ to induce insulin resistance, impede insulin secretion, and produce marked hyperglycemia [38], and in diabetic humans [39]. We are not aware of prior reports of corneal nerve density in ZDF rats. However, there was no corneal nerve loss in the equivalent db/db mouse model of type 2 diabetes [16]. It is plausible that normal or elevated corneal nerve density in these models results from hyperinsulinemia in the earlier stages of diabetes despite the decrease to similar levels as control in the later stage, as insulin has direct neuritogenic effects on sensory nerves [34] and prevents corneal nerve loss when applied to the eye of insulin-deficient diabetic mice [21]. Hyperinsulinemia may promote sprouting from terminal regions of corneal sensory nerves, as nerve density in the more proximal stromal region was not affected. That treatment with NSI-189 restored corneal nerve density towards control levels suggesting that the molecule acts as a homeostatic regulator, irrespective of whether external stressors drive fiber loss or growth.

We previously showed that NSI-189 promoted neurite outgrowth when added to cultured primary sensory neurons derived from normal and type 1 diabetic rats and also dose-dependently increased maximal and spare respiratory capacity [16], suggesting mitochondrial activation. We therefore extended our investigations to tissue obtained from diabetic rats treated with NSI-189 in vivo. DRG isolated from ZDF rats showed reduced phosphorylation of AMPK, a gate-keeper of mitochondrial function [7]. This is consistent with prior reports in rodents with type 1 or type 2 diabetes [25]. NSI-189 treatment of ZDF rats restored AMPK activity without impacting hyperglycemia. This is similar to the efficacy profiles of resveratrol [25] and muscarinic antagonists [40] in DRG derived from diabetic rodents and suggests that suppression of AMPK pathway activity can be overridden. The beneficial effect of NSI-189 on AMPK activation in DRG was accompanied by improvement of peripheral neuropathy, and although we cannot ascribe causality to this association, it is consistent with the hypothesis that NSI-189, via activation of AMPK, supports maintenance and restoration of sensory neuron homeostasis.

In contrast to multiple descriptions of peripheral neuropathy in ZDF rats [26, 27], assessments of CNS function in this model are limited to a study using the Morris water maze test after 8 weeks of untreated diabetes that reported impaired swimming velocity, possibly reflecting general physiological status, but normal learning abilities [41]. In the present study, we used the object recognition test, a test that does not rely on sustained physical activities [17] that are challenging for obese rats [41]. Impaired short-term memory in ZDF rats was accompanied by reduced expression of synaptic proteins and is consistent with reports of impaired synaptic plasticity in the brain of other rodent models of type 1 and type 2 diabetes [42–44]. Supporting its procognitive properties in both animal models [16, 23, 45] and humans with MDD [14, 15], NSI-189 treatment reversed an established memory deficit in ZDF rats and efficacy was maintained for 12 weeks. This was accompanied by protection or normalization of hippocampal CA3 volume and a significant increase in expression of synaptic plasticity marker proteins. While insulin treatment ameliorated synaptic plasticity and water maze learning deficits in type 1 diabetic rats, efficacy required treatment from onset of diabetes [43]. The ability of NSI-189 to reverse cognitive dysfunction is notable as it reproduces the most likely scenario for clinical use of a therapeutic to treat this complication of long-term diabetes.

The ability of insulin or aerobic exercise to attenuate impaired synaptic plasticity in diabetic rodents has been ascribed to enhanced insulin signaling, AMPK pathway activity, and mitochondrial function in the brain [43, 44]. Given the ability of NSI-189 to protect AMPK in the DRG of these ZDF rats, we anticipated similar efficacy in the CNS. However, in contrast to the significant reduction of AMPK phosphorylation detected in the DRG, pAMPK in the cortex was not perturbed. We are not aware of any prior report of pAMPK in the brain of ZDF rats relative to control rats, although pAMPK is reduced in brain of type 1 diabetic rats [46]. The less severe hyperglycemic environment of the CNS relative to the DRG of the ZDF rat and the high metabolic demand of cortical neurons may prevent the development of local nutrient stress. Assessment of mitochondrial protein expression and activity in the cortex of ZDF rats showed deficits that agreed with other models of diabetes. For example, decreased complex I and IV activity is consistent with previous studies in type 1 diabetic rats [47]. In the LCR rat model of prediabetes, complex III activity was significantly reduced and was associated with impaired short-term spatial memory [48], consistent with encephalopathy observed in patients with complex III deficiency [49]. Complex II/III and complex IV activities were also reduced in a previous study of the ZDF rat brain [50]. We also found a decrease in protein levels of the complex III protein and significantly decreased complex IV activity in our longer-term type 2 diabetes rat model. Treatment with NSI-189 in an intervention paradigm significantly increased expression of complex III and complex V proteins along with complex I and complex IV activity in parallel with amelioration of impaired short-term memory. These results suggest that NSI-189 is able to reverse memory impairment associated with type 2 diabetes via activation and/or enhanced expression of mitochondrial complexes I, III, IV, and V. As pAMPK is not impaired in this model, the pathways by which NSI-189 regulates the mitochondrial respiratory chain remain to be determined.

## 5. Conclusions

We extended the therapeutic potential of NSI-189 to peripheral and central neuropathy in a rat model of type 2 diabetes. Our data are consistent with the presence of mitochondrial disorders that contribute to diabetes-induced impaired peripheral [33, 51] and cerebral [52] neuronal function and degeneration. We also identified a novel potential mechanism of action for NSI-189, via regulation of mitochondrial function to maintain cellular homeostasis, with efficacy mediated at distinct points in PNS and CNS tissue. The capacity of NSI-189 to reverse multiple indices of established peripheral neuropathy and cognitive dysfunction supports the translational viability of this molecule.

## Data Availability

The data used to support the findings of this study are available from the corresponding author upon reasonable request.

## Supplementary Materials

Supplementary 1 Supplemental Table 1S shows significant obesity, hyperglycemia, hyperinsulinemia, and impaired glucose tolerance in ZDF diabetic rats. These parameters were not affected by 16 weeks of daily treatment with NSI-189.

Supplementary 2 Supplemental Figure 1S shows the results of the glucose tolerance test performed prior to treatment and after 12 weeks of treatment with NSI-189, showing insulin resistance in ZDF rats with or without treatment with NSI-189.
