# Supplementary material for: Enhancement of Mitochondrial Function by the Neurogenic Molecule NSI-189 Accompanies Reversal of Peripheral Neuropathy and Memory Impairment in a Rat Model of Type 2 Diabetes
Source: J Diabetes Res. 2022 Jul 4;2022:8566970. doi: 10.1155/2022/8566970 (PMC9372526; doi:10.1155/2022/8566970)
Supplement: Supplementary 1 — Supplemental Table 1S shows significant obesity, hyperglycemia, hyperinsulinemia, and impaired glucose tolerance in ZDF diabetic rats. These parameters were not affected by 16 weeks of daily treatment with NSI-189. [file 8566970.f1.pdf]

	**Body weight at onset of treatment (g)**	**Body weight after 16 weeks of treatment (g)**	**Blood glucose at onset of treatment (mg/dl)**	**Blood glucose after 16 weeks of treatment (mg/dl)**	**Plasma insulin at onset of treatment**	**Plasma insulin after 16 weeks of treatment (µg/l)**
**Control+Vehicle**	387.4±3.6	425.0±4.8	110.1±3.5	126.2±3.2	0.298±0.075	0.427±0.082
**ZDF + Vehicle**	455.3±10.1 ***	400.7±5.8	452.4±11.9 ***	503.3±24.2 ***	1.944±0.472 ***	0.297±0.078
**ZDF + NSI-189**	465.1±10.9 ***	407.9±10.1	439.5±13.5 ***	499.3±24.1 ***	2.016±0.296 ***	0.271±0.053
