# Supplementary figures and images for: Enhancement of Mitochondrial Function by the Neurogenic Molecule NSI-189 Accompanies Reversal of Peripheral Neuropathy and Memory Impairment in a Rat Model of Type 2 Diabetes
Source: J Diabetes Res. 2022 Jul 4;2022:8566970. doi: 10.1155/2022/8566970 (PMC9372526; doi:10.1155/2022/8566970)

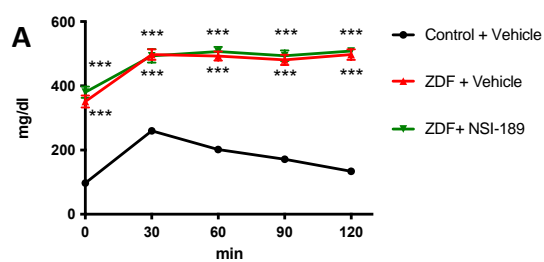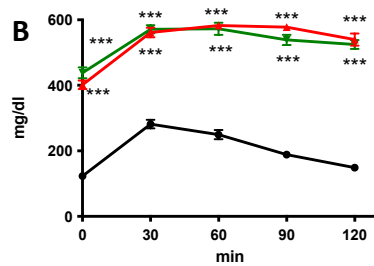

Supplement: Supplementary 2 — Supplemental Figure 1S shows the results of the glucose tolerance test performed prior to treatment and after 12 weeks of treatment with NSI-189, showing insulin resistance in ZDF rats with or without treatment with NSI-189. [file 8566970.f2.pdf]
